# Aminoguanidine affects systemic and lung inflammation induced by lipopolysaccharide in rats

**DOI:** 10.1186/s12931-019-1054-6

**Published:** 2019-05-22

**Authors:** Saeideh Saadat, Farimah Beheshti, Vahid Reza Askari, Mahmoud Hosseini, Nema Mohamadian Roshan, Mohammad Hossein Boskabady

**Affiliations:** 1 Neurogenic Inflammation Research Center, Mashhad University of Medical Sciences, Mashhad, 9177948564 Iran; 2 Department of Physiology, School of Medicine, Mashhad University of Medical Sciences, Mashhad, 9177948564 Iran; 3 Neuroscience Research Center, Torbat Heydariyeh University of Medical Sciences, Torbat Heydariyeh, Iran; 4 Department of Physiology, School of Paramedical Sciences, Torbat Heydariyeh University of Medical Sciences, Torbat Heydariyeh, Iran; 5 Student Research Committee, Department of Pharmacology, Faculty of Medicine, Mashhad University of Medical Sciences, Mashhad, Iran; 6 Department of Pathology, School of Medicine, Mashhad University of Medical Sciences, Mashhad, Iran

**Keywords:** Aminoguanidine, Inflammation, Lipopolysaccharide, Lung injury, Nitric oxide

## Abstract

**Background:**

Nitric oxide is a mediator of potential importance in numerous physiological and inflammatory processes in the lung. Aminoguanidine (AG) has been shown to have anti-inflammation and radical scavenging properties. This study aimed to investigate the effects of AG, an iNOS inhibitor, on lipopolysaccharide (LPS)-induced systemic and lung inflammation in rats.

**Methods:**

Male Wistar rats were divided into control, LPS (1 mg/kg/day i.p.), and LPS groups treated with AG 50, 100 or 150 mg/kg/day i.p. for five weeks. Total nitrite concentration, total and differential white blood cells (WBC) count, oxidative stress markers, and the levels of IL-4, IFN-γ, TGF-β1, and PGE2 were assessed in the serum or bronchoalveolar lavage fluid (BALF).

**Results:**

Administration of LPS decreased IL-4 level (*p* < 0.01) in BALF, total thiol content, superoxide dismutase (SOD) and catalase (CAT) activities (*p* < 0.001) in BALF and serum, and increased total nitrite, malondialdehyde (MDA), IFN-γ, TGF-β1 and PGE2 (*p* < 0.001) concentrations in BALF. Pre-treatment with AG increased BALF level of IL-4 and total thiol as well as SOD and CAT activities (*p* < 0.05 to *p* < 0.001), but decreased BALF levels of total nitrite, MDA, IFN-γ, TGF-β1, and PGE2 (*p* < 0.01 to *p* < 0.001). AG treatment decreased total WBC count, lymphocytes and macrophages in BALF (*p* < 0.01 to *p* < 0.001) and improved lung pathological changes including interstitial inflammation and lymphoid infiltration (*p* < 0.05 to *p* < 0.001).

**Conclusions:**

AG treatment reduced oxidant markers, inflammatory cytokines and lung pathological changes but increased antioxidants and anti-inflammatory cytokines. Therefore, AG may play a significant protective role against inflammation and oxidative stress that cause lung injury.

## Background

Bacterial lipopolysaccharide (LPS), also termed endotoxin, has shown pro-inflammatory activities [1]. LPS is present as a contaminant in cigarette-smoke, air pollution and organic dusts [2]. Average ambient air LPS concentration was measured at ±0.4 ng/m^3^ [3]. LPS inhalation stimulates the innate immune system in healthy human subjects and results in an acute lung and systemic inflammation. [3]. The consequences of these inflammatory responses include overproduction of nitric oxide (NO), tissue injury and organ failure [4]. It has been demonstrated that LPS leads to lung injury [5–8]. Chronic exposure of animals to LPS has been also shown to induce pathological features of COPD, such as pulmonary inflammation and airway hyperresponsiveness as well as structural changes in the lung [3, 9–12]. Follow-up studies have shown that long-term LPS exposure resulted in pulmonary function decline and a major lung inflammatory response. However, the extent of inflammatory processes in lung pathology of these patients is still unclear [3].

NO, a potentially toxic free radical and physiological messenger, has a major role in the regulation of the immune system functions [13] including aggregation of platelets, rolling and migration of leukocytes, and expression of inflammatory cytokines such as interleukin-1 (IL-1), interleukin-6 (IL-6), interleukin-8 (IL-8), interferon gamma (INF-γ) and tumor necrosis factor-alpha (TNF-α) [14]. During the inflammation process, endotoxins and cytokines induced rapid alterations in NO gene expression leading to the de novo synthesis of the inducible isoform of nitric oxide synthases (iNOS) and cyclooxygenase (COX-2) pathways. There are interrelated and the cross-talk between these two pathways which play a key role in the regulation of the inflammatory processes [13].

In several animal models of lung injury, inflammation and oxidative stress are involved as the underlying pathophysiological mechanisms. Thus, anti-inflammatory or antioxidant agents have been widely used to alleviate lung injury [6]. Aminoguanidine (AG), an iNOS inhibitor, affects several enzyme systems [15]. Inhibition of NO, arachidonic acid metabolites and cytokines production can be advantageous in the systemic and lung inflammation. AG was prepared more than 100 years ago [16], but relatively less attention has been paid to its beneficial effects on the respiratory system. It has been suggested that enhanced generation of NO by iNOS may contribute to acute lung injury [17]. Therefore, the present study set up to evaluate the role of inhibition of NO production by administration of AG on LPS-induced chronic systemic inflammation and oxidative stress in a rat lung injury model.

## Methods

### Animals

Fifty male Wistar rats (240 ± 10 g) were purchased from the Animal House, Mashhad University of Medical Sciences and were housed in Plexiglas cages under controlled temperature (22 ± 2 °C), humidity (54 ± 2%), and 12 h light/dark cycle. Food and water were freely available during the study period. The study was approved by the ethics committee of Mashhad University of Medical Sciences for Animal Experiments (code 951071).

### Experimental groups

Rats were randomly divided into five groups (*n* = 10 in each group) as follows: (1) Control group received saline instead of LPS and AG, (2) LPS group received LPS 1 mg/kg/day [18] for 5 weeks, (3–5) LPS groups treated with 50, 100 or 150 mg/kg/day AG 30 min before LPS injection, during 5 weeks. LPS and AG (Sigma-Aldrich Chemical Co) freshly dissolved in sterile warm saline before injection and administrated intra-peritoneally (i.p.).

At the end of the experiment, all animals were anesthetized by urethane (1.6 g/kg). To prepare blood serum, 5 ml blood was collected from the animal’s heart after opening the chest in the test tube and centrifuged at 3500 rpm for 10 min. The serum samples were collected and stored at − 70 °C for measurement of the levels of nitrite, malondialdehyde (MDA), total thiol content, superoxide dismutase (SOD) and catalase (CAT) activity [18].

### Bronchoalveolar lavage fluid (BALF) preparation

The chest was opened and the left lung was clamped to preserve architecture for histological studies. A cannula was placed into the trachea and the right lung was washed with one mL normal saline for five times (totally, 5 ml) through a tracheal cannula according to previous studies [18]. BALF was centrifuged at 2500 rpm at 4 °C for 10 min. The supernatant was collected and stored at − 70 °C for measurement of the levels of nitrite and cytokines, and assessment of oxidative stress.

### Measurement of total nitrite concentration

Total nitrite concentration was measured in the serum and BALF by Griess reagent method using a standard enzyme-linked immunosorbent assay (ELISA) kit (Promega Corp., USA, Cat#G2930). In brief, 100 μl of serum or BALF were added to a 96-well flat-bottomed microplate. Then, sulfanilamide solution and N-1-naphtylethylenediamine dihydrochloride under acidic conditions were added to all collected samples, respectively. The absorbance was detected by a microplate reader (Biotek, USA) at 520–550 nm wavelengths. The limit detection was 2.5 μM nitrite [19].

### Total and differential white blood cell (WBC) counts

Total leukocyte was determined in duplicate using a hemocytometer (in a Burker chamber). For differential WBC count, a smear was prepared from the cell pellet in BALF and blood sample and stained with Wright-Giemsa. After staining, differential count was carried out by standard morphologic protocol under the light microscope.

### Assessment of oxidative stress markers

MDA, a biological marker of lipid peroxidation, was assayed in the serum and BALF based on the reaction between MDA and thiobarbituric acid (TBA) as described previously [20]. Total thiol content was also assayed in the serum and BALF using a previous established method [20]. Here, 5, 5′-dithiobis-(2-nitrobenzoic acid) (DTNB) interact with SH groups, forming a highly colored anion with the maximum peak at 412 nm. MDA and Total thiol contents were expressed as μM.

SOD activity was assayed in the serum and BALF according to the previously described method [20]. The method is based on the generation of superoxide through auto-oxidation of pyrogallol and dependent revived inhibition of 3-(4,5-dimethyl-thiazol-2-yl) 2,5-diphenyl tetrazolium bromide (MTT) to formazan [20]. CAT activity was assayed based on its ability to decompose hydrogen peroxide (H_2_O_2_), which is reflected in the reduction of absorption at 240 nm [20]. SOD and CAT activities were expressed as units per ml.

### Measurement of IL-4, IFN-γ, TGF-β1, and PGE2 in BALF

Specific ELISA kits (ebioscience Co, San Diego, CA, USA) and the instructions provided by the manufacturer were used to measure interleukin-4 (IL-4), interferon-gamma (IFN-γ), transforming growth factor-beta-1 (TGF-β1), and prostaglandin-E2 (PGE2) in BALF. The measured absorbance of the samples in a microplate reader (Biotek, USA) was compared with an established standard curve in the same measurement, and the cytokines concentrations were calculated.

### Lung histopathological evaluation

Histological examination was performed on left lung which was not lavaged. The left lung was fixed in 10% buffered formalin (37%, Merck, Germany) and embedded in paraffin blocks. The specimens were cut into 4 μm slices and were stained with hematoxylin-eosin (H&E) solution. The tissues were then evaluated under a light microscope. The pathologic changes in the lung of different groups were included: interstitial inflammation and lymphoid infiltration. The scoring system of pathological changes was: 0, no pathologic changes; 1, patchy changes; 2, local changes; 3, scattered changes; 4, severe changes (in the most parts of the lung) [21].

### Statistical analysis

All results were considered as mean ± SEM. The percent change in all measured parameters in the LPS compared to control and in LPS-AG treated compared to untreated LPS groups were also calculated. The data were analyzed using one-way analysis of variance (ANOVA) followed by Tukey’s multiple comparison test. Statistically significant was considered as *p* < 0.05.

## Results

### Total nitrite concentration

**Fig. 1:**
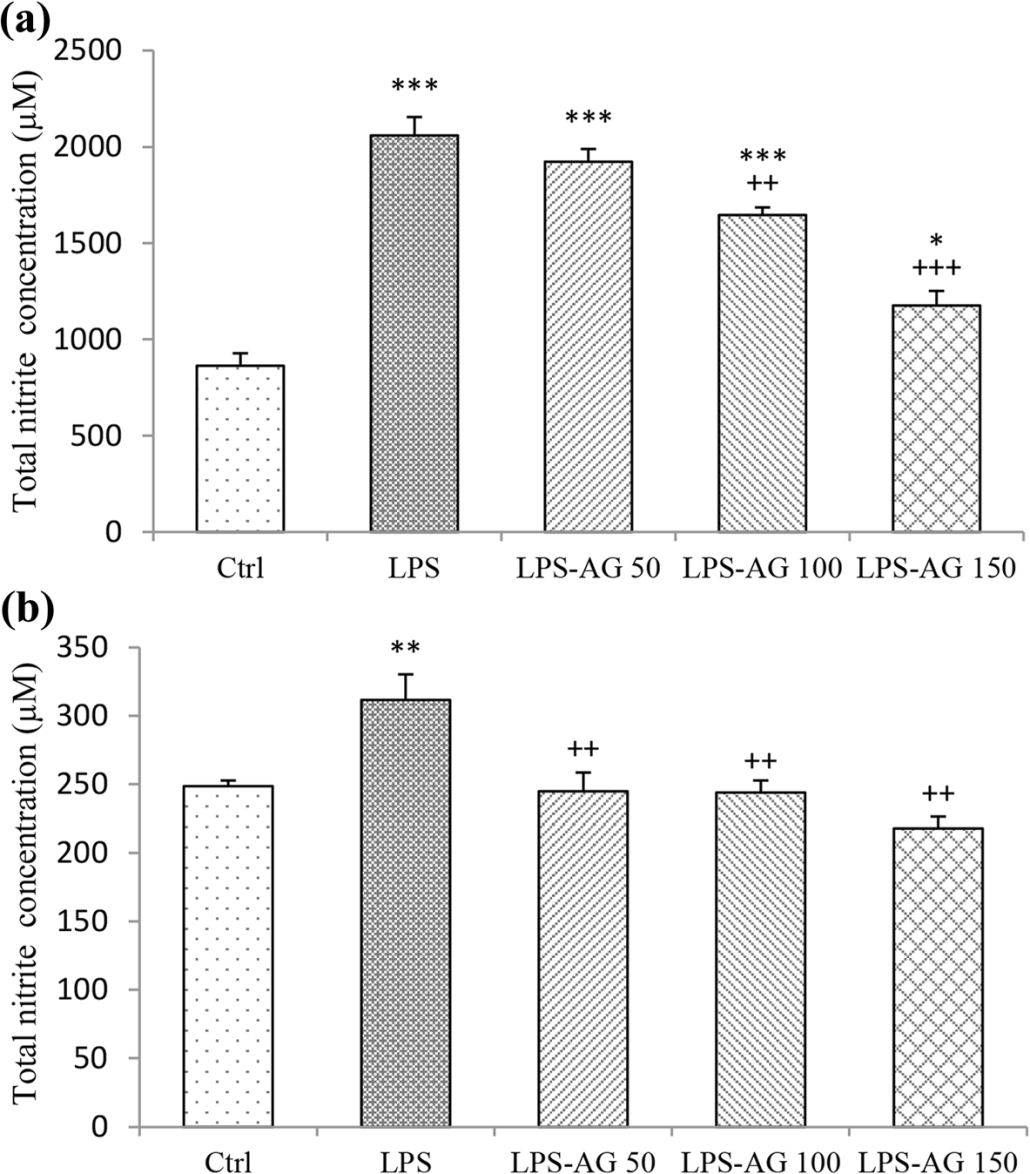
Total nitrite concentration in the serum (**a**) and BALF (**b**). Data are shown as mean ± SEM (*n* = 10 per group). *;*P* < 0.05, **;*P* < 0.01 and ***;*P* < 0.001 compared to control group, ++;*P* < 0.01 and +++;*P* < 0.001 compared to LPS group. Statistical analysis were performed using one-way analysis of variance (ANOVA) followed by Tukey’s multiple comparison test

The serum and BALF nitrite levels of LPS groups were increased by 238% (2060.91 ± 93.23) and 125% (311.63 ± 18.72), respectively relative to control group (864.7 ± 62.89 and 248.46 ± 4.24, for serum and BALF), (*P* < 0.001 and *P* < 0.01, respectively), (Table and Fig. 1). In AG treated groups with doses of 100 and 150 mg/kg, serum nitrite concentrations were reduced to 79% (1646.70 ± 39.41) and 57% (1175.90 ± 75.69), respectively relative to LPS group (2060.91 ± 93.23), (*P* < 0.01 and P < 0.001, respectively), (Table 1 and Fig. 1a). Additionally, pre-treatment with all doses of AG decreased BALF total nitrite concentration to 78% (244.83 ± 13.71), 78% (243.92 ± 8.82) and 69% (217.93 ± 8.42), respectively relative to LPS group (311.63 ± 18.72), (all, *P* < 0.01), (Table 1 and Fig. 1b).

**Table 1:** Percent changes of various measured parameters in LPS relative to control and in AG treated relative to LPS groups (%)

Parameters	Sample	LPS/Ctrl	AG/LPS		
			50	100	150
Total nitrite concentration	Serum	238	93	79	57
	BALF	125	78	78	69
Total WBC count	Blood	141	91	85	77
	BALF	182	97	86	73
Neutrophils	Blood	138	87	83	80
	BALF	126	100	92	95
Lymphocytes	Blood	142	93	86	75
	BALF	175	97	88	74
Monocytes	Blood	155	88	82	73
Macrophages	BALF	320	96	78	56
Eosinophils	Blood	109	108	100	91
	BALF	150	85	100	83
MDA concentration	Serum	240	86	62	53
	BALF	623	71	68	44
Total thiol content	Serum	26	106	162	232
	BALF	16	86	124	368
SOD activity	Serum	10	292	370	585
	BALF	13	295	468	528
CAT activity	Serum	33	133	165	214
	BALF	8	263	398	583
IL-4	BALF	42	101	155	222
IFN-γ	BALF	293	82	70	45
TGF-β1	BALF	281	86	73	46
PGE2	BALF	301	79	68	42
Interstitial inflammation	Lung tissue	732	100	90	63
Lymphoid infiltration	Lung tissue	666	79	75	49

### Total and differential WBC counts

**Fig. 2:**
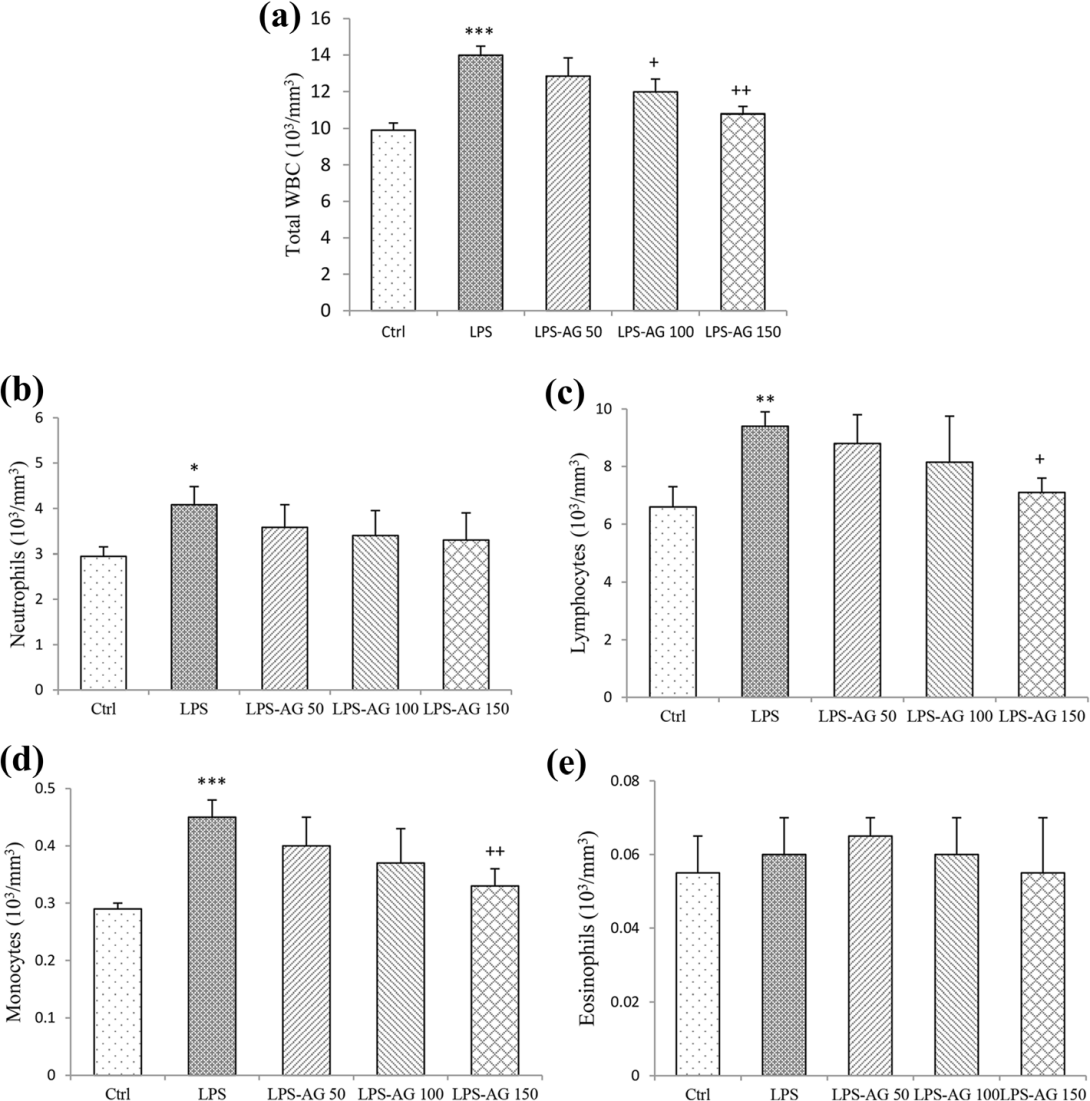
Total and differential WBC counts in the blood. Data are shown as mean ± SEM (*n* = 10 per group). *;*P* < 0.05, **;*P* < 0.01 and ***;*P* < 0.001 compared to control group, +;*P* < 0.05 and ++;*P* < 0.01 compared to LPS group. Statistical analysis were performed using one-way analysis of variance (ANOVA) followed by Tukey’s multiple comparison test

The results showed that total WBC count in the blood was increased by 141% (13.99 ± 0.50) in LPS group relative to control group (9.895 ± 0.40), (*p* < 0.001) which was due to the increased numbers of neutrophils, lymphocytes and monocytes by 138% (4.08 ± 0.40), 142% (9.4 ± 0.50) and 155% (0.45 ± 0.03), respectively relative to control groups (2.95 ± 0.20, 6.6 ± 0.70 and 0.29 ± 0.01, for neutrophils, lymphocytes and monocytes respectively), (*p* < 0.05 to *p* < 0.001, Table 1, Fig. 2).

In the treated groups with three doses of AG, total WBC counts were reduced to 91% (12.845 ± 1.00), 85% (11.98 ± 0.70) and 77% (10.785 ± 0.40) relative to LPS group (13.99 ± 0.50), (Table 1) which were significant at doses of 100 and 150 mg/kg (p < 0.05 and *p* < 0.01, respectively; Fig. 2a). In AG treated groups with three doses of AG, neutrophils count was reduced to 87% (3.58 ± 0.50), 83% (3.4 ± 0.55) and 80% (3.3 ± 0.60), lymphocytes count was reduced to 93% (8.8 ± 1.00), 86% (8.15 ± 1.60) and 75% (7.1 ± 0.50), monocytes count was reduced to 88% (0.4 ± 0.05), 82% (0.37 ± 0.06) and 73% (0.33 ± 0.03), and eosinophils count was reduced to 91% (0.05 ± 0.01), only by 150 mg/kg dose of AG relative to LPS groups (4.08 ± 0.40, 9.40 ± 0.50, 0.45 ± 0.03 and 0.06 ± 0.01, for neutrophils, lymphocytes, monocytes and eosinophils respectively), (Table 1) which were statistically significant at dose of 150 mg/kg for mononuclear leukocytes (*p* < 0.05 for lymphocyte cells and *p* < 0.01 for monocyte cells; Fig. 2b-d).

Total WBC count in BALF was increased by 182% (6.36 ± 0.50) in LPS group relative to control group (3.49 ± 0.40), (*p* < 0.001) which was due to the increased lymphocytes and macrophages counts in BALF of LPS groups by increased 175% (3.50 ± 0.30) and 320% (1.60 ± 0.10), respectively relative to control groups (2.00 ± 0.20 and 0.50 ± 0.05, for lymphocytes and macrophages), (all, *p* < 0.001).

**Fig. 3:**
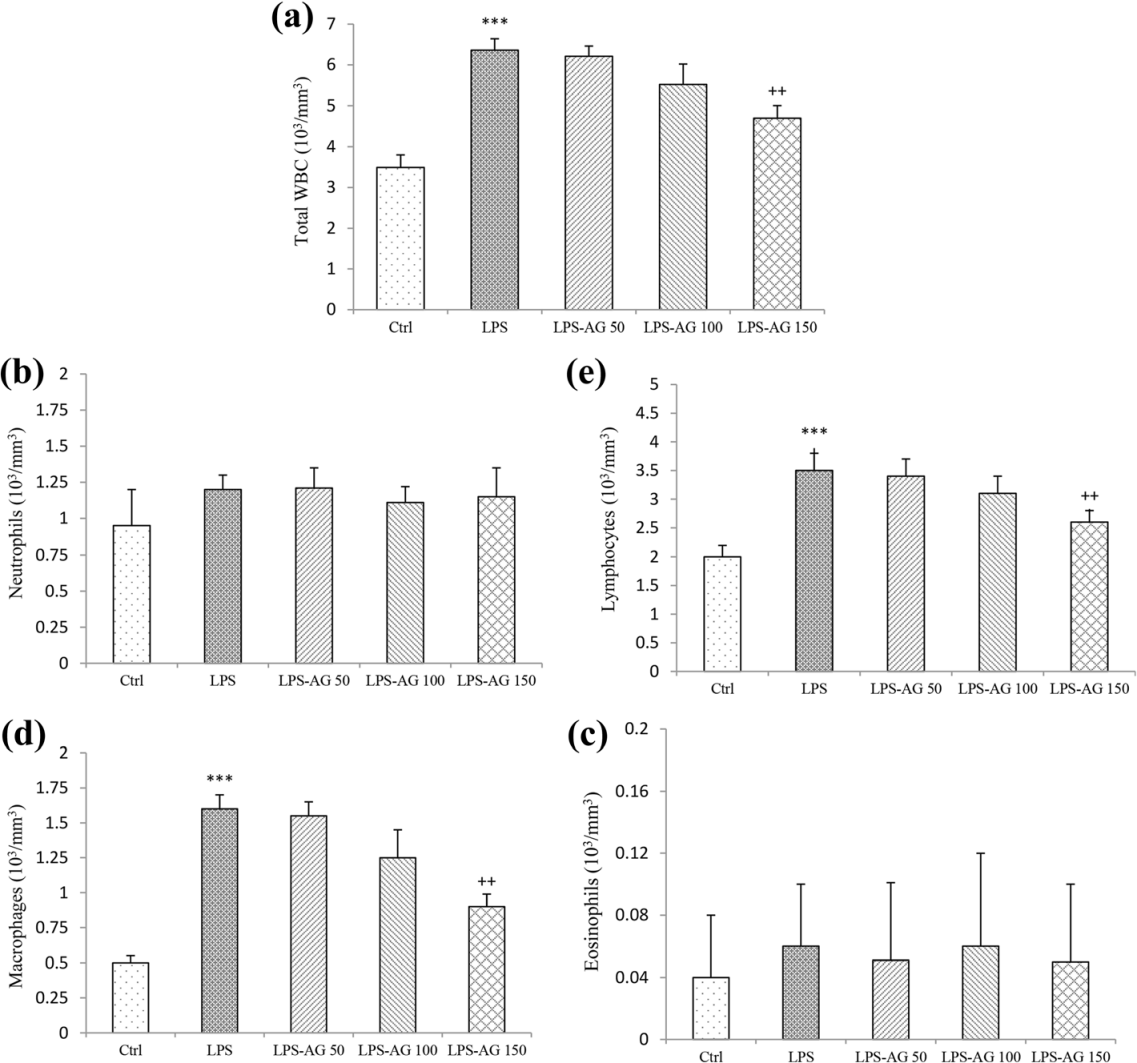
Total and differential WBC counts in BALF. Data are shown as mean ± SEM (n = 10 per group). ***;*P* < 0.001 compared to control group, ++;*P* < 0.01 compared to LPS group. Statistical analysis were performed using one-way analysis of variance (ANOVA) followed by Tukey’s multiple comparison test. The reason of unchanged neutrophil count in BALF of LPS group is unknown to us

In the treated groups with three doses of AG, total WBC counts in BALF were reduced to 97% (6.21 ± 1.00), 86% (5.52 ± 0.70) and 73% (4.70 ± 0.40), respectively relative to LPS group (6.36 ± 0.50), (Table 1) which was significant at AG 150 mg/kg (*p* < 0.01; Fig. 3).

In AG treated groups, lymphocytes count was reduced to 97% (3.40 ± 0.30), 88% (3.10 ± 0.30) and 74% (2.60 ± 0.20), and macrophages count was reduced to 96% (1.55 ± 0.10), 78% (1.25 ± 0.20) and 56% (0.90 ± 0.09), due to its three doses respectively, relative to LPS group (1.60 ± 0.10), (Table 1) which were significant for AG 150 mg/kg (both, *p* < 0.01; Fig. 3).

### Oxidant marker (MDA) content

**Fig. 4:**
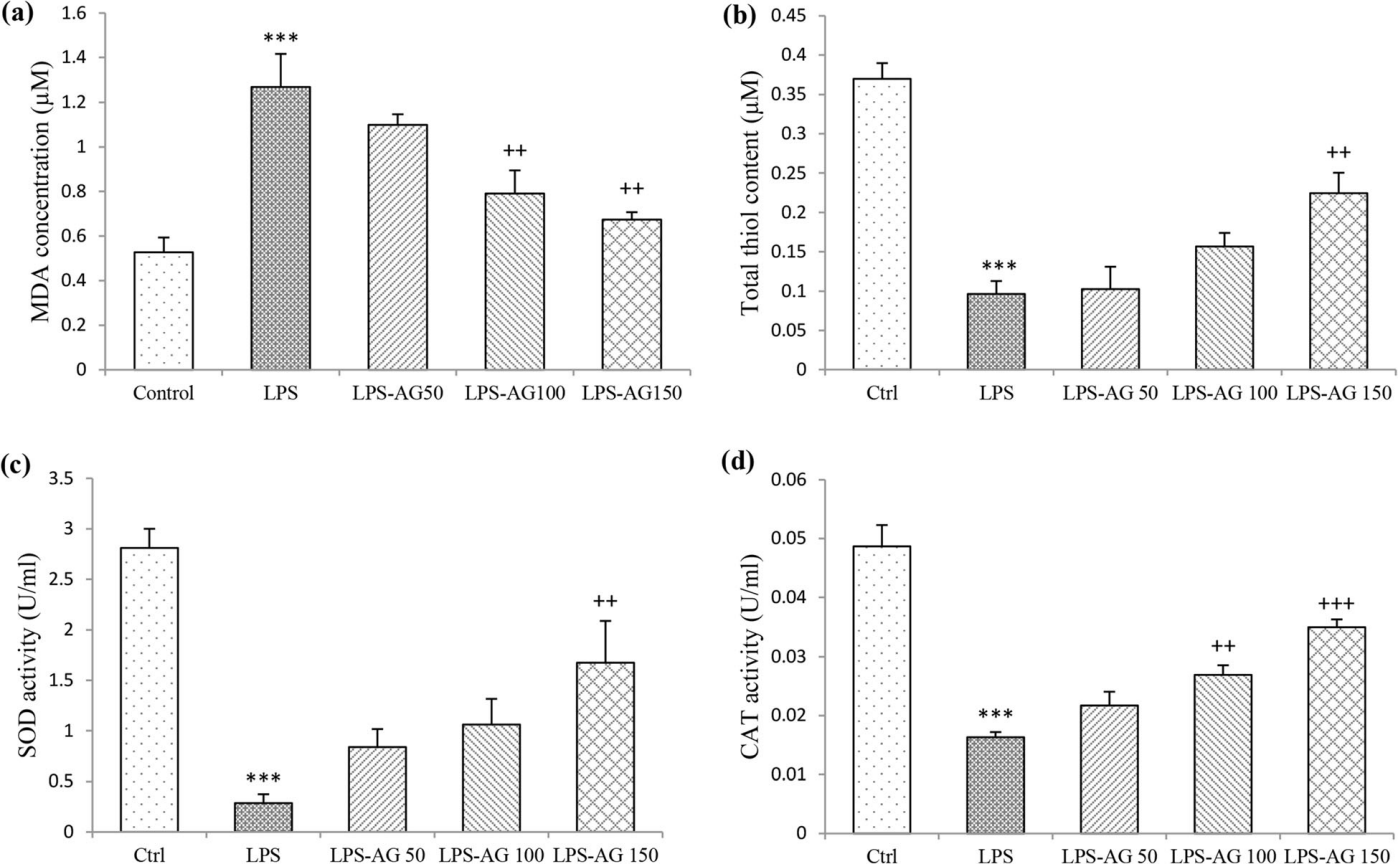
MDA concentration (**a**), thiol content (**b**), SOD (**c**) and CAT activities (**d**) in the serum. Data are shown as mean ± SEM (*n* = 10 per group). ***;*P* < 0.001 compared to control group, ++;*P* < 0.01 and +++;*P* < 0.001 compared to LPS group. Statistical analysis were performed using one-way analysis of variance (ANOVA) followed by Tukey’s multiple comparison test

**Fig. 5:**
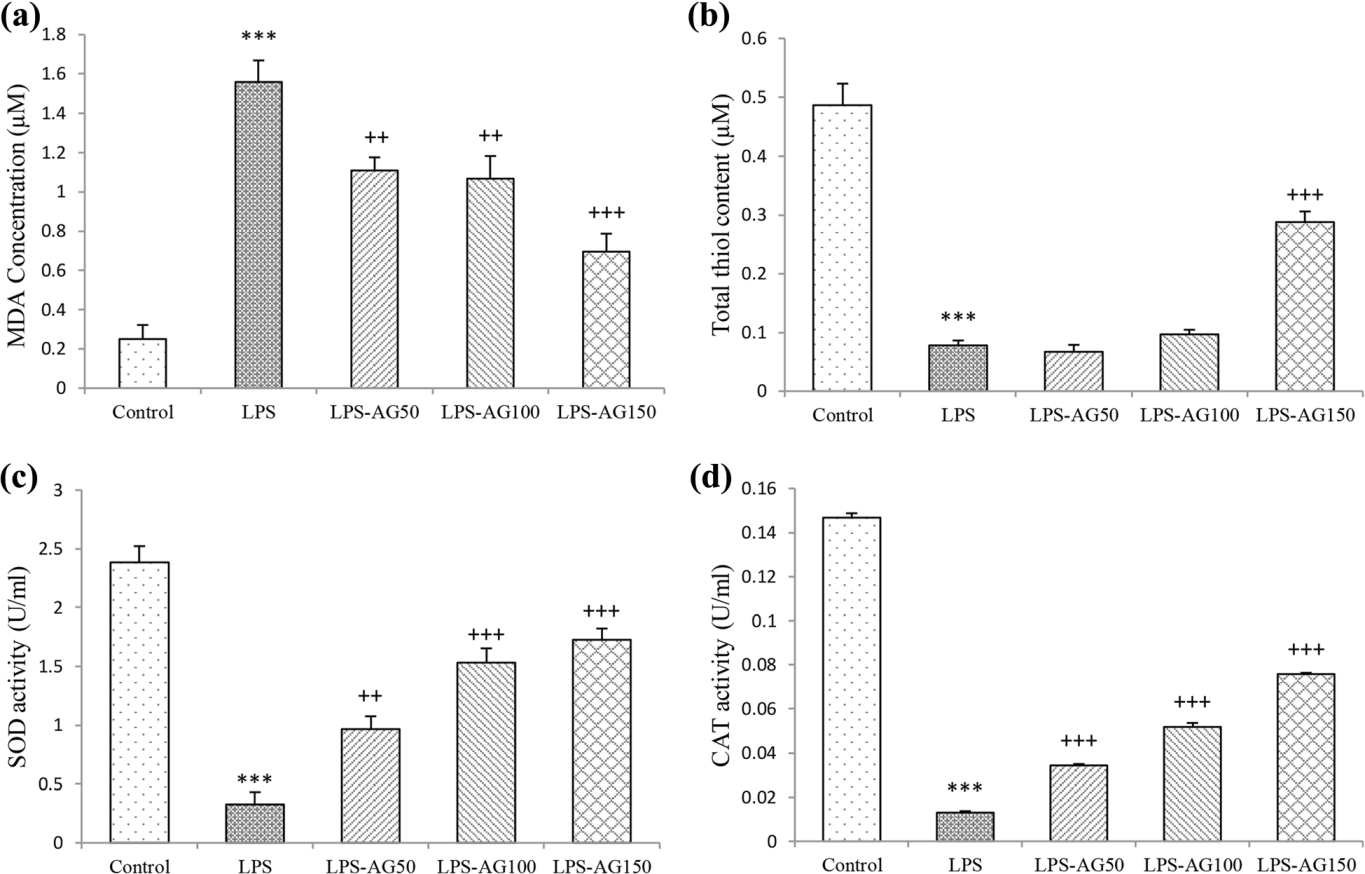
MDA concentration (**a**), thiol content (**b**), SOD (**c**) and CAT activities (**d**) in BALF. Data are shown as mean ± SEM (*n* = 10 per group). ***;*P* < 0.001 compared to control group, ++;*P* < 0.01 and +++;*P* < 0.001 compared to LPS group. Statistical analysis were performed using one-way analysis of variance (ANOVA) followed by Tukey’s multiple comparison test

Serum and BALF MDA concentrations of LPS group were increased by 240% (1.26 ± 0.14) and 623% (1.55 ± 0.11), respectively relative to control groups (0.52 ± 0.06 and 0.25 ± 0.07, for Serum and BALF), (both, *p* < 0.001). In AG treated groups, serum MDA concentrations were reduced to 62% (0.79 ± 0.10) and 53% (0.67 ± 0.03) relative to LPS group (1.26 ± 0.14), (*p* < 0.01 for AG 100 and 150 mg/kg). BALF MDA concentration was reduced to 71% (1.10 ± 0.06), 68% (1.06 ± 0.11) and 44% (0.69 ± 0.09), respectively by three doses of AG relative to LPS group (1.55 ± 0.11), (p < 0.01 to *p* < 0.001), (Figs. 4a and 5a, and Table 1).

### Anti-oxidant markers (thiol, SOD and CAT)

In LPS group, total thiol content, SOD and CAT activities in the serum were reduced to 26% (0.09 ± 0.01), 10% (0.28 ± 0.08) and 33% (0.01 ± 0.00), respectively, relative to control group (0.36 ± 0.02, 2.80 ± 0.18 and 0.04 ± 0.00, for total thiol content, SOD and CAT respectively), (all, *p* < 0.001). Additionally, total thiol content, SOD and CAT activities in BALF were reduced to 16% (0.07 ± 0.00), 13% (0.32 ± 0.10) and 8% (0.01 ± 0.00), respectively, relative to control groups (0.48 ± 0.03, 2.38 ± 0.13 and 0.14 ± 0.00, for total thiol content, SOD and CAT respectively), (all, *p* < 0.001), (Fig. 5b-d and Table 1).

In the serum of AG 150 mg/kg treated group, total thiol content was increased by 232% (0.22 ± 0.02), and SOD activity was increased by 585% (1.67 ± 0.41), (both, *p* < 0.01). Pre-treatment with AG 100 and 150 mg/kg increased CAT activity to 165% (0.02 ± 0.00) and 214% (0.03 ± 0.00), (p < 0.01 and *p* < 0.001, respectively), (Fig. 4b-d and Table 1). In BALF of AG treated groups, SOD activity was increased by 295% (0.96 ± 0.10), 468% (1.53 ± 0.12) and 528% (1.72 ± 0.09), and CAT activity was increased by 263% (0.03 ± 0.00), 398% (0.05 ± 0.00) and 583% (0.07 ± 0.00), (*p* < 0.01 to *p* < 0.001), relative to LPS groups (0.32 ± 0.10 and 0.01 ± 0.00, for SOD and CAT). Pre-treatment with AG 150 mg/kg increased total thiol content by 368% (0.28 ± 0.01), relative to LPS group (0.07 ± 0.00), (*p* < 0.001), (Fig. 5b-d and Table 1).

### Levels of IL-4, IFN-γ, TGF-β1, and PGE2 in BALF

**Fig. 6:**
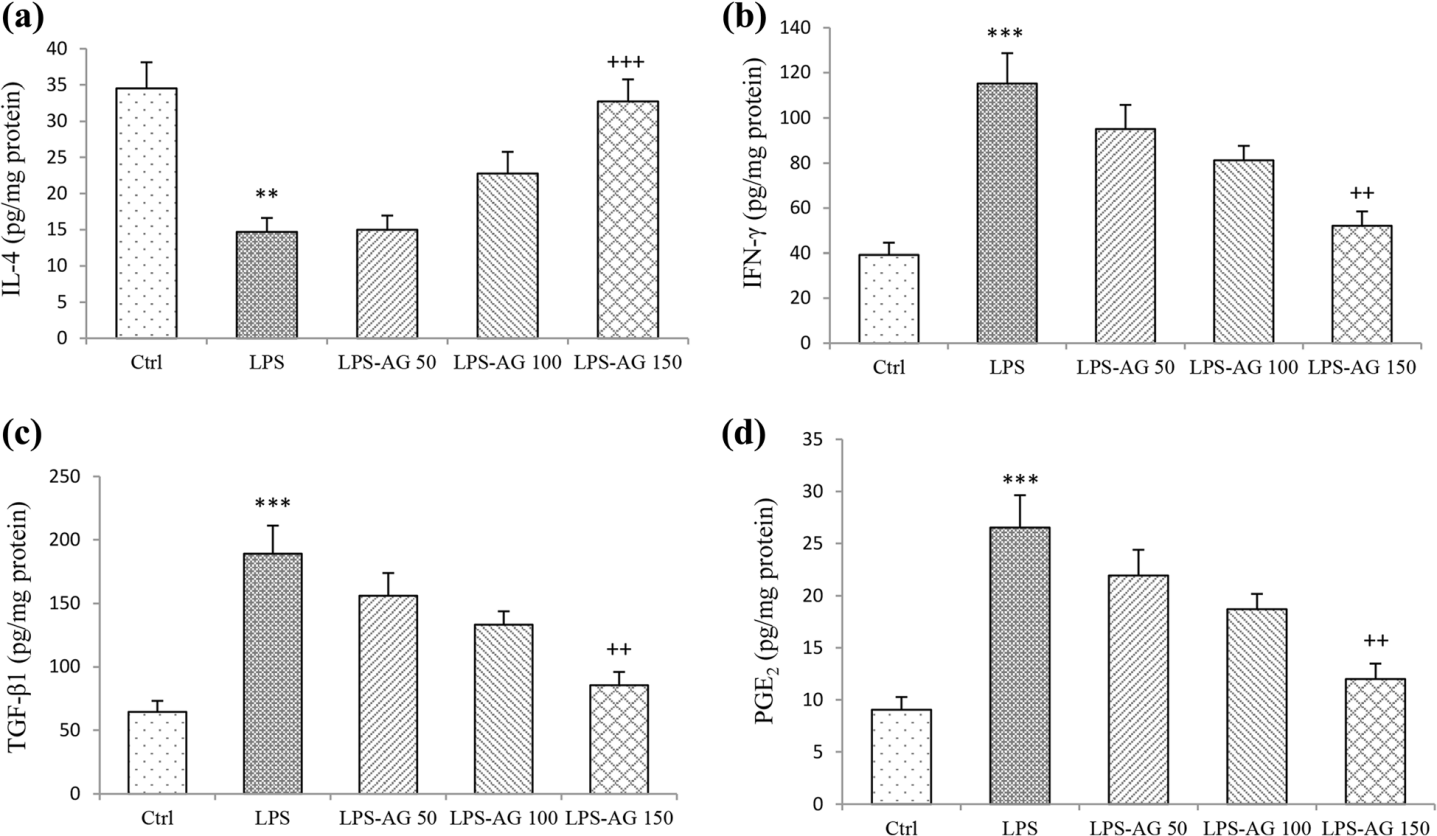
The levels of IL-4 (**a**), IFN-γ (**b**), TGFβ1 (**c**), and PGE2 (**d**) in BALF. Data are shown as mean ± SEM (n = 10 per group). **;*P* < 0.01 and ***;*P* < 0.001 compared to control group, ++;*P* < 0.01 and +++;*P* < 0.001 compared to LPS group. Statistical analysis were performed using one-way analysis of variance (ANOVA) followed by Tukey’s multiple comparison test

In LPS group, BALF level of IL-4 was reduced to 42% (14.69 ± 1.93), (*p* < 0.01), and the levels of IFN-γ, TGF-β1 and PGE2 were increased to 293% (115.15 ± 13.51), 281% (189 ± 22.17) and 301% (26.52 ± 3.11), respectively, relative to control groups (34.55 ± 3.57, 39.25 ± 5.31, 64.42 ± 8.71 and 9.04 ± 1.22, for IL-4, IFN-γ, TGF-β1 and PGE2 respectively) (all, *p* < 0.001), (Fig. 6a-d and Table 1). In AG 150 mg/kg treated group, IL-4 level was increased to 222% (32.75 ± 3.00), (p < 0.001), while IFN-γ level reduced to 45% (52.06 ± 6.43), TGF-β1 level was reduced to 46% (85.45 ± 10.55), and PGE2 level was reduced to 42% (11.99 ± 1.48), relative to LPS groups (14.69 ± 1.93, 115.15 ± 13.51, 189 ± 22.17 and 26.52 ± 3.11, for IL-4, IFN-γ, TGF-β1 and PGE2 respectively), (all, *p* < 0.01), (Fig. 6a-d and Table 1).

### Lung histopathological evaluation

**Fig. 7:**
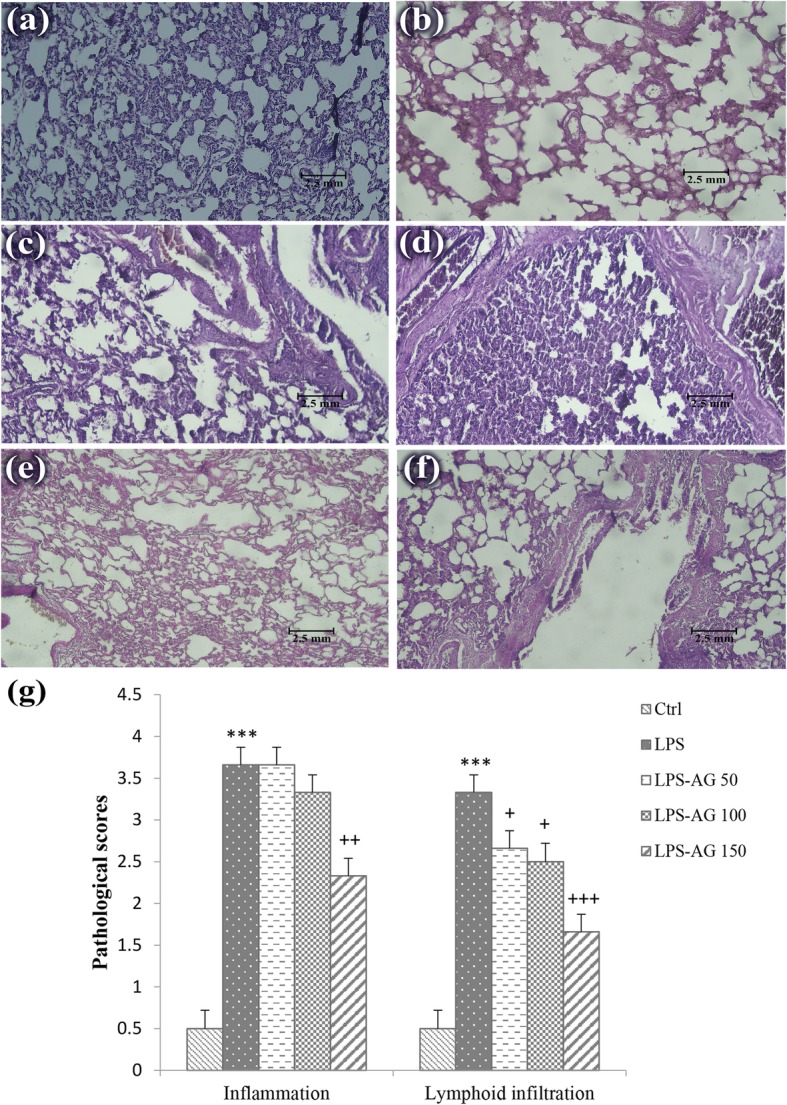
Photographs of a lung specimen in control lung tissues (**a**), interstitial inflammation and lymphoid infiltration in LPS (**b** and **c**), AG50 (**d**), AG100 (**e**) and AG150 (**f**) groups (magnification for each group; 10 × 20; scale bar; 2.5 mm). The degree of lung injury was measured using lung pathological score (**g**). ***;*P* < 0.001 compared to control group, +;*P* < 0.05, ++;*P* < 0.01 and +++;*P* < 0.001 compared to LPS group. Data are shown as mean ± SEM. The lung pathological changes were scored as follows: 1) no pathologic changes, 0; 2) patchy changes, 1; 3) local changes, 2; 4) scattered changes, 3 and 5) severe changes (in the most parts of the lung), 4. Statistical analysis were performed using one-way analysis of variance (ANOVA) followed by Tukey’s multiple comparison test

Pathological changes in the LPS group, including the interstitial inflammation and lymphoid infiltration were increased to 732% (3.66 ± 0.21) and 666% (3.33 ± 0.21), respectively relative to control group (0.5 ± 0.22 for the interstitial inflammation and lymphoid infiltration), (*p* < 0.001 for both cases; Fig. 7 and Table 1).

In the treated groups, interstitial inflammation was reduced to 63% (2.33 ± 0.21) and lymphoid infiltration was reduced to 79% (2.66 ± 0.21), 75% (2.5 ± 0.22) and 49% (1.66 ± 0.21), respectively, relative to LPS group (3.66 ± 0.21 and 3.33 ± 0.21, for the interstitial inflammation and lymphoid infiltration), (Table 1) which were statistically significant at doses of 50 and 100 mg/kg for lymphocyte infiltration (*p* < 0.05) and 150 mg/kg for interstitial inflammation and lymphoid infiltration (*p* < 0.01 and *p* < 0.001, respectively).

## Discussion

The results of the present study showed that chronic i.p. administration of LPS for five weeks has led to an increase in total nitrite concentration, and WBC count as well as elevated monocytes, lymphocytes and neutrophils counts in the blood and lung lavage. LPS also induced oxidative damage by increasing MDA concentration and decreasing total thiol concentration as well as SOD and CAT activities in the serum and lavage. Decreased IL-4 level and increased IFN-γ, TGF-β1, PGE2 levels in the lung lavage were also observed due to chronic LPS administration. At five weeks after LPS administration, severe pathological changes including interstitial inflammation and lymphoid infiltration were also observed.

Previous studies showed that long-term LPS exposure induced various types of pulmonary diseases which characterized by chronic inflammatory processes in the lung [3]. In acute lung injury, a major component of the inflammatory response is infiltration of activated neutrophils into the lung [22]. Animal experiments have demonstrated bronchoalveolar neutrophilia being the most prominent cell response following bacterial LPS inhalation [23]. LPS inhalation in healthy subjects increased neutrophils and lymphocytes levels in BALF [23]. Acute LPS exposure increased neutrophil count in BALF in both rabbits [24] and rats [25]. Increased neutrophils count in BALF of mice was detected 1 h post LPS inhalation which was persisted for 48 h [26]. In the present chronic lung injury model of LPS exposure, there was no significant increase in neutrophil count in BALF. However, the results indicated increased total WBC in the blood by 141% which was due to increased monocytes, lymphocytes and neutrophils counts but in the lavage by 182% which was accompanied by increased macrophage and lymphocytes. The reason of unchanged neutrophil count in BALF of LPS group is unknown to us.

LPS can activate neutrophils and macrophages to produce reactive oxygen species (ROS) [27] which lead to the production of inflammatory mediators such as generation of diverse pro-inflammatory Th1 type cytokines including IFN-γ [28] as well as inflammatory cytokines and chemokines which recruit more neutrophils to tissues exposed by LPS, and propagate the inflammation process [27–29]. IL-4 also caused substantial reductions in neutrophil content in BALF [30]. Thus, inhibition of iNOS can be helpful in reducing systemic and lung inflammation.

It was shown that TGF-β suppress the macrophage response to LPS, in vitro and decreased systemic inflammation [31] and plays an important role in epithelial changes, sub-epithelial fibrosis, airway smooth muscle remodeling, and microvascular changes [32]. Previous studies also showed increased serum levels of TGF-β in LPS-induced inflammation [31], which is in agreement with results of the current study.

Elevated IL-1β, IL-4, IL-6 and IFN-γ levels in lung tissue, one hour after administration [33] and increased IL-1β mRNA, IL-10 mRNA, and IL-4 protein at one hour after LPS challenge in the lung of mice [34] were reported. Down-regulation IL-10, and up-regulation off TNF-α, IL-6, IL-4 and IL-1β production in the BALF [35] as well as a significant up-regulation in the gene expression of TNF-α, IL-1β, IL-6 and IL-12, and a down-regulation in the gene expression of IL-4 and IL-10 were observed in LPS-induced acute lung injury in vivo and in vitro [36]. In LPS three-hit model of rat acute lung injury induced by LPS (1.5 mg/kg) injected into the endotracheal following by i.p. injection of LPS (3 mg/kg) and then endotracheal administration of LPS (3 mg/kg) 48 h later, the expression of TNF-α and IFN-γ was first enhanced but declined thereafter. The results of the above mentioned studies were in line with the results of the present study. Therefore, LPS induces Th1 responses (IFN-γ) and inhibits Th2 responses through the TLR4-dependent pathway that triggers the activity of NOS-II [37].

PGE2 could modulate the activity of NOS by the direct effect of TNFα on the release of NO from macrophages or synergic effect of TNFα with IFN-γ [38]. In a rat acute lung injury model, intratracheal administration of LPS reduced the content of arachidonic acid in blood neutrophils and increased the level of PGE2 in BALF [25]. The elevated level of PGE2 following administration of LPS may have a protective role in the lungs, but its function may depend on acute or chronic nature of inflammatory response.

LPS three-hits can induce rapid pulmonary fibrosis which the first rapid pulmonary fibrosis stage occurred on days 3–7, whereas from 14 to 21 days was the second stage [39]. Acute infusion of LPS (5 mg/kg over 60 min) in rabbits caused extensive morphologic lung damage [24]. Chronic LPS exposure can cause neutrophil-dependent emphysematous changes in lung architecture and result in other pulmonary changes such as airway wall thickening, mucus cell metaplasia, irreversible alveolar enlargement, and the chronic inflammatory response [3, 10, 40]. Therefore, the inflammatory and pathologic changes were similar with lung pathological changes in chronic inflammatory lung diseases, especially COPD patients, suggesting that this murine model could be applicable to the pathogenesis of COPD condition [3].

Treatment with AG resulted in a decrease in total nitrite concentration and WBC count. AG modified oxidative status by decreasing the levels of MDA and increasing total thiol content as well as SOD and CAT activities both in serum and BALF of LPS-AG groups. Increased IL-4 level and decreased IFN-γ, TGF-β1, PGE2 levels in BALF of AG treated was also observed. The pathological changes in the lung tissues including interstitial inflammation and lymphoid infiltration were also improved due to AG treatments dose-dependently.

The inhibitory effect of AG on iNOS was reported in a dose-dependent manner [41] in various conditions such as in rat model of hemorrhagic shock [42, 43] and in LPS-induced increased NO production in the primary culture of rat hepatocytes [15]. In the present study, the similar pattern was observed for the inhibitory effect of AG on nitrite level in the serum and BALF of chronic LPS exposure-induced lung injury. Acute infusion of AG (1 mg/kg one hour after the end of LPS infusion) in rabbits decreased neutrophil count in BALF [24]. However, this effect was not observed in the LPS-induced blood neutrophilia in the present study. This contradiction may be related to the type of inflammation or may be due to the acute and chronic effects of AG. The present findings suggest that AG probably reduces up-regulation of iNOS by decreasing alveolar macrophages. The relationships between COX-2 and iNOS isoforms were previously reported [44]. AG can reduce the production of NO and PGE2 induced by LPS injection and affected the PG metabolism by inhibiting COX-2 expression and its activity [45]. The current findings suggest that iNOS-mediated NO production could result in lung damage and this could lead to up-regulation of COX-2, which increases the production of ROS and toxic prostanoids.

AG is able to scavenge hydroxyl and peroxyl radicals [46] in various conditions including experimental model of diabetes mellitus which it reduced the levels of pulmonary oxidative stress and increased collagen synthesis and deposition in the lung [47] as well as against a single dose of paraquat-induced oxidative stress in the lung of mice [48] which support the results of the current study.

AG also preserved lung function and shifted the Th2 to the Th1 response with a reduction of IL-4 and IL-13 and increase in IL-1β production in ovalbumin sensitized animals [49] which caused increase IL-4 level unlike LPS-induced inflammation. Reduction of glomerular iNOS and TGF-β1 mRNA expression in mice and rats models of glomerulosclerosis and diabetic nephropathy by AG were also reported [50, 51] which were in line with the results of lung injury induced by LPS of the present study. The absence of the effect of AG on in vivo expression of TNFα and IL-1β in the lungs of endotoxemic rats was reported [41] but there are reports on the effect of AG in the serum and tissue cytokine levels [41]. However, the effect of AG on BALF level of cytokine and oxidant, anti-oxidant has not been studied extensively.

Histological examinations showed a reduction in kidney, liver, lung, and brain damages for AG [42]. It is suggested that the treatment of rabbits with infusion of AG, attenuated acute lung injury and inflammation following intravenous exposure to LPS [24]. AG also prevented bleomycin-induced lung fibrosis in both rats and mice [52, 53] which were in agreement with the present findings in the chronic lung injury by LPS. Due to the role of TGF-β in most of the biological processes leading to the airway remodeling, reduced BALF level of TGF-β1 can lead to improved pathological changes in the lung tissue.

There are no findings about the possible mechanisms of AG in chronic lung injury induced by LPS. Based on the results of this study, potential mechanisms of AG may be via dual inhibition of NO and PGE2, enhanced production of IL-4, as a strong anti-inflammatory cytokine, which in turn decreased inflammatory cytokines, IFN-γ and TGF-β1, as well as monocyte chemotaxis. The radical scavenging properties of AG may help to explain the modulation of lung and systemic inflammation by this compound.

Although the protective effects of AG on lung disorders was examined in several previous studies as listed above, the unique novelty of the present study is evaluating of the effect of AG on chronic lung injury induced by LPS administration which is an endotoxin-induced lung injury model. However, there are some similarities between chronic endotoxin-induced lung injury model and COPD [3]. In addition, LPS is present as a contaminant in cigarette-smoke [2] the main cause of COPD. Therefore, AG could be also a potential therapeutic candidate for treatment of COPD which should be examined in further studies.

There was no any mortality among studied animals of different groups but body weight changes was not evaluated which should be evaluated in further studies. In fact, both animal and human studies indicated very low toxicity of AG. However, high doses of AG are associated with some adverse effects such as autoimmune symptoms, abnormal liver function, gastrointestinal disturbance, and flu-like symptoms [54, 55]. In the present study, the left lungs were removed and placed into 10% buffered formalin for lung pathological evaluation and the right lung was washed with normal saline for preparation of BALF samples and the pro-inflammatory mediator levels were measured in BALF according the previous studies [18, 56]. It is well-known that evaluation of all parameters in a single study is impossible. Therefore, quantify steady state mRNA levels of the proinflammatory molecules and oxidative stress/antioxidative stress related genes were not evaluated in the present study and should be examined in further studies. Similarly, in vitro experiments for the involvement of specific cell type in systemic inflammation and lung injury induced by chronic LPS administration should be also examined in further studies.

## Conclusion

The present study showed that AG modulated immune and inflammatory responses in chronic lung injury by LPS administration. Therefore, AG has a protective role in LPS-induced lung injury caused by inflammation and oxidative stress. The results of the present study suggest that inhibition of iNOS by AG may be effective in the treatment of systemic and lung inflammation by both decreasing the nitrite level and/or possibly the involvement of cytokines.

## Abbreviations

AG: Aminoguanidine
BALF: Bronchoalveolar lavage fluid
CAT: Catalase
COPD: Chronic obstructive pulmonary disease
COX: Cyclo-oxygenase
DTNB: 5, 5′-dithiobis-(2-nitrobenzoic acid)
IL-1: Interleukin-1
IL-4: Interleukin-4
IL-6: Interleukin-6
IL-8: Interleukin-8
INF-γ: Interferon gamma
iNOS: Inducible nitric oxide synthase
LPS: Lipopolysaccharide
MDA: Malondialdehyde
MTT: 3-(4,5-Dimethylthiazol-2-yl)-2,5-diphenyltetrazolium bromide
NO: Nitric oxide
NOS: Nitric oxide synthase
PGE2: Prostaglandin-E2
ROS: Reactive oxygen species
SOD: Superoxide dismutase
TBA: Thiobarbituric acid
TGF-β1: Transforming growth factor-beta-1
TNF-α: Tumor necrosis factor-alpha
WBC: White blood cell
